# Lipidomic Insight into Eggs and Meat of Quail (*Coturnix japonica*) as Potential ‘Superfoods’

**DOI:** 10.3390/molecules31030407

**Published:** 2026-01-24

**Authors:** Małgorzata Białek, Wiktoria Wojtak, Marian Czauderna, Kamil Zaworski, Agnieszka Białek

**Affiliations:** 1 The Kielanowski Institute of Animal Physiology and Nutrition, Polish Academy of Sciences, Instytucka 3, 05-110 Jabłonna, Poland or w.wojtak@ifzz.pl (W.W.); m.czauderna@ifzz.pl (M.C.); k.zaworski@ifzz.pl (K.Z.); a.bialek@ifzz.pl (A.B.); 2 Institute of Animal Science, Warsaw University of Life Sciences, Ciszewskiego 8 Street, 02-786 Warsaw, Poland; 3 School of Health and Medical Sciences, VIZJA University, Okopowa 59, 01-043 Warsaw, Poland

**Keywords:** egg, fatty acids, meat, storage, superfoods, quail

## Abstract

Background/Objectives: The rising global demand for sustainable and nutritionally valuable food sources highlights the importance of exploring alternatives to conventional livestock. Japanese quail (*Coturnix japonica*) have gained attention as an environmentally efficient species, offering high-quality eggs and meat with favorable nutritional profiles. This study aimed to characterize the fatty acid (FA) composition of quail eggs (QEs) and meat (QM), investigating breast (B) and thigh (T) muscles produced in Poland under small-scale farming conditions, with a focus on assessing their functional foods’ potential. Methods: Gas chromatography–mass spectrometry analysis was applied to determine FA profiles in fresh and stored samples. Statistical evaluation included two-way ANOVA, principal component analysis (PCA), and cluster analysis. Results: Results demonstrated that QE contained the highest total FA levels, dominated by monounsaturated fatty acids, with notable contributions from long-chain n-3 polyunsaturated fatty acids (PUFAs) and conjugated linoleic acid isomers. In contrast, QM were characterized by higher proportions of PUFA, with significant differences between breast and thigh samples. Storage influenced the levels of selected FA, particularly n-3 PUFA and the n-3/n-6 ratio. PCA and cluster analysis confirmed clear separation between eggs and muscles, regardless of storage status. Overall, both QE and QM exhibited a favorable PUFA/SFA ratio, exceeding dietary recommendations. Conclusions: These findings underscore the nutritional and functional value of quail-derived foods, supporting their consideration as sustainable alternatives to chicken products and potential candidates for the ‘superfood’ category.

## 1. Introduction

Due to the constant growth of the global population and shrinking natural resources, there is an increasing awareness of the need to use resources more efficiently, conserving them for future generations. Sustainable farming practices are becoming increasingly important nowadays, as people are reflecting on the impact they have on the environment and are looking for ways to decrease collective carbon footprint and help to reduce the growing threat of climate change. Sustainable food sources directly enrich the health and nutritional value of food supply by reducing exposure to harmful chemicals and pollutants. Growing awareness of food security, environmental impact, and animal welfare make people seek alternative options to traditional livestock. Quail, a small game bird, has gained popularity as a sustainable food source in recent years [1]. Japanese quails (*Coturnix japonica*, JQ) possess several advantages, including small body size, rapid growth, high productivity, and relatively low rearing costs, making them well-suited to both large-scale commercial operations and smallholder systems [2]. As consumers in developed countries increasingly demand high-quality food produced under environmentally sustainable conditions, producers face the dual challenge of meeting growing food needs while simultaneously reducing the ecological footprint of intensive production systems. In this context, quail farming offers a promising solution, as it is associated with lower greenhouse gas emissions and a reduced carbon footprint [3].

Currently, JQ are bred for both scientific and commercial purposes, as they are excellent animal models for biomedical experiments and, at the same time, are valued for high production of eggs with high nutritional value, and their delicate meat. Worldwide, in the European Union (EU) and in Poland, quails are mostly used for egg production rather than meat. According to FAOSTAT data, quails make up about 11.8% of the world’s domestic bird population in 2018. Quail egg (QE) production is about 1.3 million tons per year, which is almost 10% of global egg production. Quail meat (QM) production accounts for only about 0.2% of global poultry meat production, which translates to 200,000–240,000 tons per year. On a global scale, QE production is the most important in Asia, with China as the largest producer of QE, accounting for over 35–40% of the global production. In the EU, the production of QE and QM mainly concentrates in France, Italy, and Spain [4].

Quail carcasses are characterized by a high proportion of breast muscles and low adipose tissue content. Quail meat has a characteristic taste that predominates red fibers over light ones. Compared to chicken meat, QM is characterized by a higher protein content (approx. 25%) of high biological quality (with about 39 g of exogenous amino acids per 100 g of protein), lower fat content (approx. 4%) and energy value (approx. 126 kcal/100 g).

The nutritional value of QE is very similar to that of hen’s eggs; however, in some respects, they may have an advantage. QEs are characterized by a higher yolk to white proportion. The calorie content of QE and chicken eggs is similar, but QE are typically consumed in lower quantities per serving due to their smaller size. QEs contain more essential exogenous amino acids and less fat. They are a rich source of iron, copper, carotene, B vitamins, and they have a high content of bioavailable phosphorus, not found in other food products. They contain more cholesterol, but less choline and vitamin D. QEs have a low glycemic index, thanks to which the level of glucose in the blood increases slowly after consumption. Due to the presence of ovomucoid, QEs are generally safe to people allergic to hen egg white. The taste of QE is more pronounced than hen’s egg, even similar to the aroma of game. The longer shelf life of QE (at room temperature about 30 days, in the refrigerator up to 3 months) is associated with less food waste during the distribution.

There are several previous foodomics studies on QM and QE, but the vast majority of them analyzed the impact of a specific factor, such as dietary modifications [5,6,7,8,9,10,11], age/degree of exploitation [12], species [13], or production type [14]. Nevertheless, only a few screening studies on the lipidomic composition of QE are available [15,16,17], while studies concerning the quality of meat available for retail sale are still scarce. That is why our research, where both egg and meat compositions are evaluated, is of great importance and necessity.

Currently, there is no universally accepted scientific definition of the term ‘superfood’, which was first introduced by Moss in Nature Nutrition in 1998 to describe exceptionally nutritious foods. Nowadays, the term is commonly used for natural, minimally processed products rich in nutrients and bioactive compounds, yet its application remains largely marketing-driven and lacks clearly defined scientific criteria. As the literature indicates, the relationship between food composition and the ‘superfood’ label is largely interpretative: although such products often contain high levels of antioxidants, vitamins, minerals, fiber, or polyunsaturated fatty acids, their composition alone is insufficient to reliably predict health effects [18,19]. Moreover, the ‘superfood’ concept often overlooks the complex interactions of nutrients within the human body, the broader dietary context, and ethical or environmental implications of production. Santunione and Montevecchi argue that the superfood narrative should integrate responsible consumption, sustainable farming, and animal welfare, distinguishing between ‘synthesizers’ and ‘accumulators’ of essential nutrients [20]. Taken together, these considerations highlight the need for comprehensive biochemical evaluation of underappreciated, nutrient-dense food sources—such as Japanese quail eggs and meat—which may hold potential for this category, though their classification requires careful scientific justification. Thus, we hypothesize that, from a lipidomic perspective, QE and QM could be considered as ‘superfoods’. For this reason, the aim of the present study was to investigate the fatty acid profiles of fresh and stored quail eggs, as well as breast and thigh muscle tissues from quails reared in Poland.

## 2. Materials and Methods

### 2.1. Sample Collection

Eggs and meat samples were kindly donated by “Ecofarming” (Łask, Poland), a small commercial farm maintaining dual-purpose production of Japanese quail (*Coturnix japonica*). Birds were kept in metal aviaries (n = 3) with wooden floor (3 m × 0.8 m × 0.5 m) (length × width × height) and fed with complete commercial feed, with no antibiotic growth promoters or hormonal substances (Piast Pasze Sp. z o.o., Lewkowiec, Poland). A total of 30 QEs were randomly collected (10 eggs per one aviary) from laying birds aged 10–12 weeks. Only clean, intact eggs with characteristic shell color were selected. The eggs were stored on trays at 4 ± 1 °C and a relative humidity of approximately 75% until transport. Meat samples were obtained from 30 quails (10 birds from one aviary, sex ratio 1:1) aged 13 weeks. The birds were slaughtered on-site under hygienic conditions compliant with Regulation (EC) No. 853/2004. After evisceration, the right pectoral (B) and right femoral (T) muscles were manually excised from each carcass, and packed individually in hermetic polyethylene bags. Within 24 h after collecting eggs and meat samples, the material was transported to the laboratory, in thermally insulated containers, maintaining a temperature between 2 and 8 °C. Upon arrival, meat was frozen and stored for 5 weeks in –20 °C. Eggs were stored for 5 weeks in 4 °C. Samples were subjected to further analyses within 24 h of arrival (‘fresh’) and after 5 weeks of storage (‘stored’). Samples of commercial feed were also delivered and subsequently analyzed.

### 2.2. Determination of Fatty Acid Profile

Fatty acids (FA) in all samples (meat, eggs, feed) were determined with the use of gas chromatography coupled with mass spectrometry (GC-MS). Prior to chromatographic analyzes, samples were subjected to alkaline hydrolysis followed by pre-column mild base- and acid-catalyzed methylations to obtain fatty acid methyl esters (FAMEs), according to Białek et al. [21]. Feed samples were grounded in the mortar, meat samples were homogenized, and the content of each egg was mixed until uniformity, and then weighed (~100 mg). Analyses were conducted on a modern chromatographic system: a Shimadzu GC-MS-QP2010 Plus EI model (Tokyo, Japan) with a quadruple mass selective detector (Model 5973 N, Shimadzu, Tokyo, Japan) on a BPX70 fused silica column (120 m × 0.25 mm i.d. × 0.25 μm film thickness; Phenomenex, Torrance, CA, USA). Helium (He) was a carrier gas, operated at a constant pressure (223.4 kPa) and flow rate of 1 mL/min. Injector and detector temperatures were 200 °C and 240 °C, respectively. The MS was operated in the EI mode and full scan monitoring (*m*/*z* 30–350). The electron energy of the ion source was equal to 70 eV. Total FA profile was determined as methyl esters with addition of nonadecanoic acid (C19:0) as the internal standard (IS). The FAME profiles in 1 μL samples at the split ratio of 10:1 were determined using the following temperature gradient program: the column was initially operated at 70 °C for 4 min, and then the temperature was increased at a rate of 12 °C/min to 150 °C, held for 6 min, increased at a rate of 8 °C/min to 168 °C, held for 27 min, increased at a rate of 0.75 °C/min to 190 °C, held for 10 min, increased at a rate of 1.8 °C/min to 210 °C, held for 15 min, increased at a rate of 6 °C/min to 234 °C, held for 4 min, increased at a rate of 6 °C/min to 236 °C, and held for 20 min [22]. The FA identification was based on electron impact ionization spectra of FAME, and compared to authentic FAME standards (Sigma, St. Louis, MO, USA) and the NIST 2007 reference mass spectra library (National Institute of Standard and Technology, Gaithersburg, MD, USA), as well as on FA retention times (obtained from TIC and SIM chromatograms). Results were expressed as μg of FA per g of sample.

### 2.3. Statistical Analysis

The Statistica (data analysis software system, version 13, TIBCO Software Inc., Palo Alto, CA, USA, (2017)) was used. Data are presented as means ± standard deviation (SD). For assessment of distribution normality, the Shapiro–Wilk test was used, and the Levene test was used for evaluation of the homogeneity of variance. The differences between sample status (fresh vs. stored) and sample type (egg vs. breast vs. thigh) and their interactions were evaluated using a two-way ANOVA. When interaction occurred (at *p* ≤ 0.05), the significances of differences were established using *post hoc* HSD RIR Tukey test (for variables with normal distribution) or a multiple comparison test (for variables with skew distribution). Differences were considered statistically significant at *p* ≤ 0.05.

Chemometric approach was also applied. Prior to the analysis, all original data were auto-scaled (standardized). Only variables that were significantly different (*p* ≤ 0.05) were subjected to analysis. Matrix of 41 variables (content of FA) was used for principal component analysis (PCA). The number of principal components (PC) was chosen using the screen test criterion. Results were presented on biplot diagram on the plane of factor projection (PC1/PC2). Cluster analysis was carried out using the agglomeration method. Euclidean distance was used as the distance determination method and the Ward method was used as the agglomeration method. To analyze similarity and create heat maps, the method of grouping features and objects was used.

## 3. Results

The detailed composition of quail feed is presented in Table S1. The most prevalent FA were c9c12 C18:2 (LA), c9c12c15 C18:3 (ALA), c9 C18:1 (OA), C16:0, and C18:0.

The total FA profiles in muscles (both B and T) differed significantly from the one in eggs (Table 1). Eggs were the richest source of FA among all analyzed samples; total content of FA was six and nearly eight times higher than these detected in breast and thigh muscles, respectively.

In the egg samples, monounsaturated fatty acids (MUFAs) were quantitatively predominant, whereas the levels of saturated (SFA) and polyunsaturated fatty acids (PUFAs) were comparable. The most abundant representatives of each FA group were oleic acid, palmitic acid, and linoleic acid, respectively. The presence of C6:0, C22:0, c7C15:1, c11C18:1, c12C18:1, c14C18:1, c11C22:1, c13C22:1, t9t12C18:2, c6c9c12c15C18:4, c9t11CLA, t10t12CLA, c11c13CLA, c11c14c17C20:3 was characteristic for the QE samples.

In the meat samples, the most abundant FA group was PUFA, followed by SFA and MUFA. In both muscle types, linoleic acid was the quantitatively dominant PUFA, whereas oleic acid was the predominant MUFA. Among SFAs, palmitic acid was the most abundant in the pectoral muscle, while stearic acid prevailed in the femoral muscle. The contents of predominant MUFA (c9C16:1, C9C18:1 (OA)), as well as PUFA (c9c12c18:2, (LA), c9c12c15 C18:3 (ALA)) were higher in B than in T samples. The FA characteristics for meat samples, not present in eggs, were the following: c9C12:1, c6c9c12C16:3, c6c9c12C18:3 (GLA), c8c11c14c17C20:4.

Significant differences between fresh and stored samples were detected in the content of C15:0, C22:0, c14C18:1, c11C22:1, c9c12c15 C18:3, c11c14c17C20:3, AA, c8c11c14c17C20:4, DHA, sum of n-3 PUFA, and n-3/n-6 ratio. The significant interactions between sample status (fresh vs. stored) and sample type (QE Vs. B vs. T) were observed for C15:0, C21:0, C22:0, c14C18:1, c11C22:1, c8c11c14C18:3, c9c12c15 C18:3, c11c14c17C20:3, and sum of n-3 PUFA.

**Figure 1:**
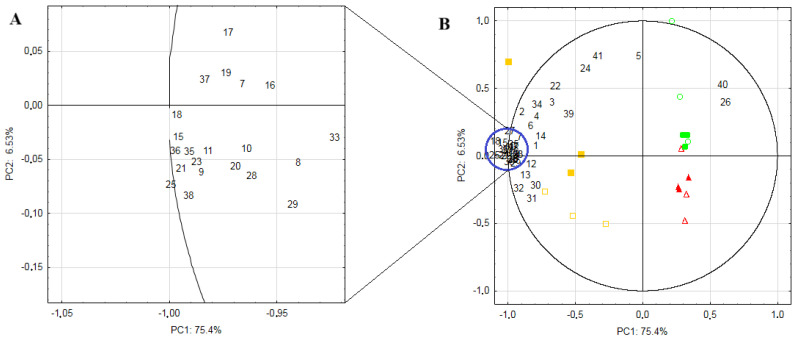
Biplot of fatty acids content in the samples obtained from quails. 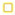 EF—fresh egg; 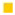 ES—stored egg; 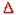 BF—fresh breast muscle; 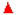 BS—stored breast muscle; 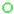 TF—fresh thigh muscle; 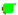 TS—stored thigh muscle; 1—C8:0; 2—C9:0; 3—C10:0; 4—C11:0; 5—C12:0; 6—C13:0; 7—C14:0; 8—C15:0; 9—C16:0; 10—C17:0; 11—C18:0; 12—C20:0; 13—C21:0; 14—C24:0; 15—ΣSFA; 16—c9C14:1; 17—c7C16:1; 18—c9C16:1; 19—c10C17:1; 20—t9C18:1; 21—c9C18:1 (OA); 22—c15C24:1; 23—ΣMUFA; 24—c9c12C16:2; 25—c9c12C18:2 (LA); 26—c6c9c12C18:3 (GLA); 27—c8c11c14C18:3; 28—c9c12c15C18:3 (ALA); 29—c11t13C18:2 CLA; 30—c11c14C20:2; 31—c8c11c14C20:3; 32—c5c8c11c14c17C20:5 (EPA); 33—c7c10c13c16c19C22:5 (DPA); 34—c4c7c10c13c16c19C22:6 (DHA); 35—ΣPUFA; 36—Total FA; 37—Σn-3; 38—Σn-6; 39—ΣSFA/ΣPUFA; 40—ΣSFA/ΣUFA; 41—Σn-3/Σn-6; ΣSFA—sum of saturated fatty acids; OA—oleic acid; ΣMUFA—sum of monounsaturated fatty acids; LA—linoleic acid; GLA—γ-linolenic acid; ALA—α-linolenic acid; CLA—conjugated linoleic acid; EPA—eicosapentaenoic acid; DPA—docosapentaenoic acid; DHA—docosahexaenoic acid; ΣPUFA—sum of polyunsaturated fatty acids; ΣUFA—sum of unsaturated fatty acids; total FA—total sum of all detected fatty acids; Σn-3 PUFA—sum of polyunsaturated fatty acids of the n-3 family; Σn-6 PUFA—sum of polyunsaturated fatty acids of the n-6 family; Σn-3PUFA/Σn-6 PUFA—ratio of n-3 polyunsaturated fatty acids to the n-6 polyunsaturated fatty acids. The overlapped part of this graph, marked by blue circle, is presented in details/enlarged at the lefts side of this figure. To facilitate understanding the Figure, enlarged part was marked with (**A**) while the main/whole figure was marked with (**B**).

Principal Component Analysis (PCA) identified 17 factors, of which the first two were selected for further analysis, carrying a total of 81.9% of the total variability (Table 2). These two PCs explained 64.3% of the total variance (PC1: 75.4% and PC2: 6.53%, respectively). PC1 is responsible for the discrimination between eggs and meat samples, as B and T samples were located at positive values of PC1, while QE samples were located in negative values of this PC. PC2 allows the discrimination between muscle types, as B is placed with negative scores, while T has high positive scores of this PC (Figure 1).

**Figure 2:**
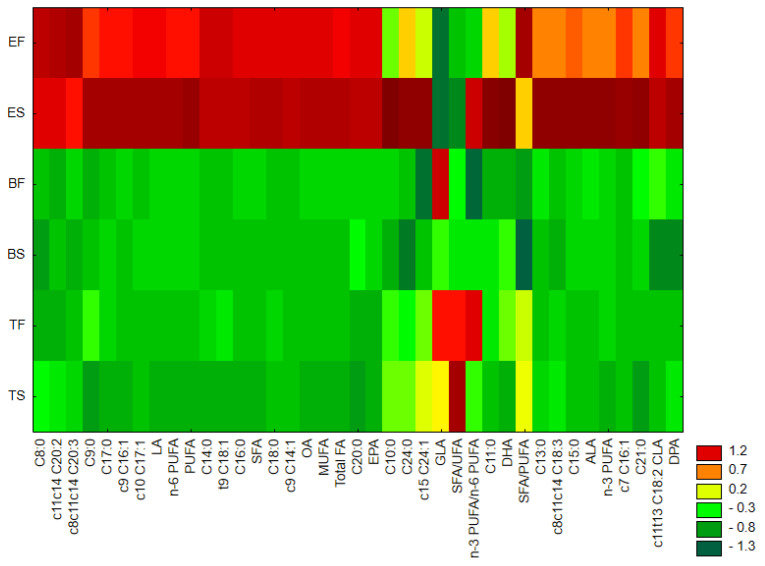
Heat map—similarity analysis performed by grouping features and objects. EF—fresh egg; ES—stored egg; BF—fresh breast muscle; BS—stored breast muscle; TF—fresh thigh muscle; TS—stored thigh muscle; SFA—sum of saturated fatty acids; OA—oleic acid; MUFA—sum of monounsaturated fatty acids; LA—linoleic acid; GLA—γ-linolenic acid; ALA—α-linolenic acid; CLA—conjugated linoleic acid; EPA—eicosapentaenoic acid; DPA—docosapentaenoic acid; DHA—docosahexaenoic acid; UFA—sum of unsaturated fatty acids; PUFA—sum of polyunsaturated fatty acids; total FA—total sum of all detected fatty acids; n-3 PUFA—sum of polyunsaturated fatty acids of the n-3 family; n-6 PUFA—sum of polyunsaturated fatty acids of the n-6 family; n-3 PUFA/n-6 PUFA—ratio of n-3 polyunsaturated fatty acids to the n-6 polyunsaturated fatty acids.

Results of chemometric analyses also confirmed that the overall FA profile of muscle samples significantly varied from that of egg. A heat map (Figure 2), prepared with the use of the method of grouping features and objects, illustrate the similarity in the levels of FAs among analyzed samples. It clearly demonstrates that the highest content of almost all detected FAs was found in egg samples, especially after storage, while muscle samples contained lower amounts of determined FA, except for GLA and the ΣSFA/ΣUFA and Σn-3PUFA/Σn-6 PUFA ratios, which levels were the highest in fresh B and T. The contents of C10:0 and DHA detected in fresh eggs samples were similar to those determined in B and T samples (both fresh and stored), while the content of c15C24:1 was similar to this detected in stored T muscle.

**Figure 3:**
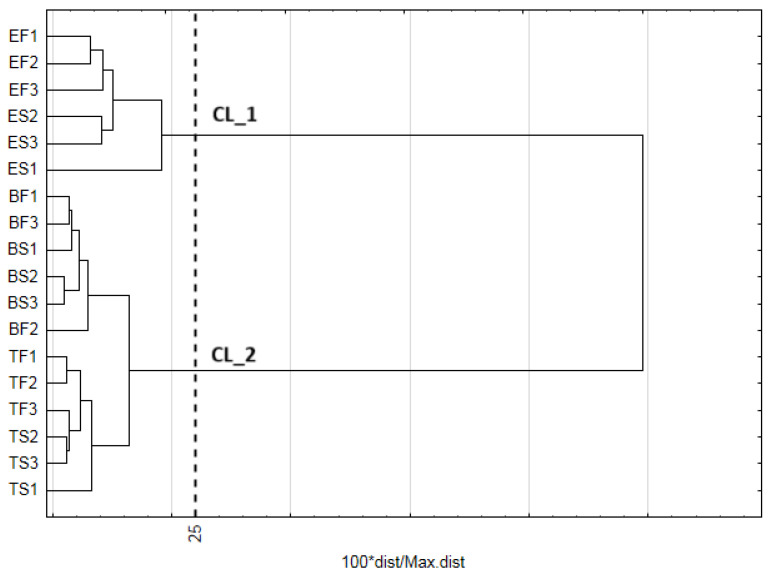
Dendrogram of similarities of examined objects (samples) based on fatty acid content. (Method of grouping: Ward agglomeration procedure; function of the distance: Euclidean distance; Mojena’s rate: d = 18.10). EF—fresh eggs; ES—stored egg; BF—fresh breast muscle; BS—stored breast muscle; TF—fresh thigh muscle; TS—stored thigh muscle.

Cluster analysis (CA), based on similarities in FA profiles of all analyzed samples, was employed to generate a dendrogram (Figure 3). By utilizing Sneath’s criterion of 33%, it resulted in the division of samples into two clusters (CL_1 and CL_2). The first cluster (CL_1) consists of egg samples, both fresh and stored. Samples of pectoral and femoral muscles formed the second cluster (CL_2), irrespective of the status (fresh or stored).

## 4. Discussion

Although there are written testimonies that the Japanese quail eggs and meat were used in eastern food and medicine from the 17th century onward, the true domestication of this bird species and its geographic spread occurred during the last century. Nowadays, they are used for production of meat and eggs. However, reliable data on domestic quail production are limited, which contributes to the underestimation of their role in poultry farming. The available data suggested that the quail population comes second after chickens in the global production of poultry meat and eggs. This clearly confirms the role of products obtained from these birds for human consumption and the need to shed more light on this food through deepened research [23].

Feeding and housing conditions have a significant impact on the composition and quality of Japanese quail eggs and meat. Birds kept in cages exhibit earlier maturation, greater efficiency, higher feed intake, and increased egg production [24]. However, further research is needed to better understand the FA profile of eggs from quails fed with a commercial diets, in order to evaluate the nutritional value of such feeding practices. Therefore, in the present study, the FA profile of eggs and meat from Japanese quails JQ reared on commercial feed and housed in aviaries was analyzed.

The study of the FA composition in eggs and meat as well as fresh and stored (under recommended storage conditions, within the shelf life) is an innovative aspect of this research, as although studies on the impact of various storage conditions on egg quality are available, studies on meat quality are scarce. Moreover, in the research, parameters such as water content, pH, proximate chemical composition, color, texture, shell quality were taken into account [25,26,27,28,29,30], while FA composition analyses lacked these aspects. Thus, our research seems to fill the substantial gap in knowledge about the FA profile of stored eggs and meat.

Cold storage can promote oxidative changes in lipids; however, in this study, differences between fresh and stored samples were minimal (Table 1). This stability can be explained by several mechanisms. The content of naturally occurring compounds of antioxidant properties, e.g., ovalbumin, ovotransferrin, phosvitin, phospholipids, vitamin E, vitamin A, selenium, and carotenoids, as well as antioxidant enzymes, e.g., glutathione peroxidase and superoxide dismutase, inhibits FA peroxidation [31,32]. Structural compartmentalization of lipids into lipoproteins, phospholipids, and triacylglycerols reduces oxygen exposure and limits oxidative propagation [33,34]. Refrigeration (4 °C) suppresses activity of lipolytic enzymes, but not completely. As it was observed by Wang et al., lipolysis and oxidation continue to occur at 4 °C, confirmed by an increase in free fatty acids (FFAs) and peroxidation product (TBARS) contents with the extension of storage time to 60 days [35]. This phenomenon may partially explain the increase in the content of total FA in eggs and thigh muscle observed in our study. In comparison to femoral muscle, there is no such correlation in the case of the pectoral muscle, where the total FA content decreases during cold storage. The stability of poultry muscle during refrigerated storage was confirmed by Eleftheriadou et al. [36].

The pectoral and femoral muscles of poultry differ in function, which is reflected in the composition of their lipid fractions. In general, oxidative (slow-twitch) muscles—thigh—exhibit higher capacity for FA oxidation, elevated mitochondrial density, and greater intramuscular fat (neutral lipids) stores compared to glycolytic (fast-twitch) muscles (breast) in many poultry species. In quails, more oxidative muscles (thigh) have a higher content of TAG and FFA than more glycolytic muscles (breast). However, unlike chickens, the breast muscle of quails is not as extremely glycolytic—its profile is more “intermediate” due to its adaptation to flight [37]. Quail pectoral muscles consist of approximately 87% red fibers and 13% white fibers, whereas leg muscles contain 45% red fibers and 55% white fibers [12].

In the study, the differences not only in the total FA content but also in the concentration of individual FA were observed in meat. The deposition of essential PUFA (LA and ALA), as well as FA arising from these precursors, namely GLA, AA, and EPA, was higher in the pectoral muscle compared to the femoral one, while the content of DHA was higher in the thigh. In chickens, by contrast, LA, ALA, and GLA were preferentially incorporated into thigh rather than breast muscle and DHA was predominantly deposited in the breast [38]. These differences may be caused by the interspecies differences in muscle fiber composition and oxidative phenotype. In broilers, the pectoralis major is composed almost exclusively of type IIB fibers, with type I and IIA fibers absent from its superficial region, whereas in quails the breast muscle contains both type IIA and IIB fibers [39,40]. The levels of those FAs, which were significantly affected by the storage, were predominantly elevated after 35 days of refrigeration in comparison to the fresh samples. The only exception was the content of C15:0 in thigh and the content of ALA in breast, which were lower after cold storage (Table S1). Variation in the content of individual FA between B and T muscles may also be a result of the differences in the activity or gene expression of key enzymes of the FA desaturation and elongation pathway. The stearoyl-CoA desaturase, responsible for the conversion of palmitic acid (C16:0) into palmitoleic acid (c9 C16:1) and stearic acid (C18:0) into OA in pectoral muscle, seems to be of higher activity or its gene expression seems to be intensified in comparison to femoral muscle, as confirmed by the elevated levels of FA being the products of its action in B samples [41]. Similarly, the gene expression and desaturating activity of FADS1 and FADS2, responsible for formation of AA, EPA, and GLA from their precursors, seem to be increased in the B samples as compared to T [42].

The study revealed distinct differences in the FA profiles of JQ eggs and muscle tissues (breast and thigh), emphasizing the nutritional/physiological uniqueness of each sample type. Eggs showed the highest total FA content among all samples, confirming their role as a dense source of lipids, particularly MUFA. These findings are consistent with previous research indicating that eggs accumulate substantial lipid reserves in the yolk to support embryonic development [43]. The FA profiles of muscle samples, in contrast to eggs, were characterized by higher PUFA content relative to SFA and MUFA—a pattern typical of avian skeletal muscle. These differences likely reflect variations in lipid turnover, enzymatic activity, and energy metabolism between yolk deposition and muscle tissue maintenance, which result from their distinct physiological roles [44].

The FA composition of eggs will be adapted to developmental requirements and the membrane structure of embryo. Essential FA, PUFA, and other vital nutrients (e.g., amino acids, antioxidants) deposited in the hatching egg constitute the initial nutritional source for the developing avian embryo. Inadequate or imbalanced nutrient provision during this early developmental window may exert long-lasting effects on offspring physiology, including impaired growth performance, altered tissue development and maturation, and compromised immune competence [45]. In muscles, FA composition can be shaped by muscle metabolism, activity, diet, and metabolic adaptations. The lipid profile of muscles is therefore determined mainly by the needs of contractile functions, energy metabolism, and muscle cell structure, rather than by the developmental or nutritional requirements of another organism [46]. Results obtained in the present study clearly indicate that sample type had greater impact on FA profile, which is explained by their different biological structure and completely different physiological purposes. However, in the present study, these two types of samples were analyzed in terms of usability in human nutrition.

Food products of quail origin due to high OA contents should be taken into consideration in terms of prevention of heart diseases [47]. The best sources of oleic acid were eggs, breast, and thigh, containing on average 7.95; 0.81, and 0.49 mg OA per g of tissue, respectively. The presence of considerable levels of long-chain n-3 PUFAs (EPA and clupanodonic acid c7c10c13c16c19 C22:5), along with conjugated linoleic acid (CLA) isomers (c9t11 C18:2, t10c12 C18:2, c11c13 C18:2), enhances the nutritional value of QE, as these compounds play key roles in immune regulation and neurodevelopment [48]. Moreover, the n-6/n-3 ratio of QE, B, and T reported in the present study (about four) was multiple times lower than this proportion claimed by other authors for quail meat (about 34.5 [47]) and chicken thigh (20 [49]). From a nutritional perspective, a minimum PUFA/SFA ratio of 0.45 is recommended for the overall diet [15]. Since this ratio exceeded the recommended level in all analyzed samples (Table 1), it can be concluded that both QE and QM—whether fresh or stored—represent valuable components of a well-balanced human diet. All these abovementioned observations seem to support the claim that QE, B, and T may be considered as ‘superfoods’.

Chemometric analysis further confirmed the biochemical differences between eggs and muscle tissues observed in the two-way statistical analysis. The clear separation along PC1 and the formation of two distinct clusters (egg vs. muscle) indicate that tissue type is the primary determinant of FA profile variability, outweighing the effect of storage or freshness. The differences between fresh and stored products were minimal, so in fact the storage process does not adversely affect the lipidomic quality of QE and QM. Therefore, in summary, it can be concluded that both products can be considered as ‘superfoods’ during their shelf life, provided they have been stored under the recommended conditions.

Notably, this study has several limitations that should be considered. First, all eggs and meat samples originated from a small-scale commercial farm, which may limit the generalizability of the findings to quails kept under different housing systems, feeding regimes, or environmental conditions. Second, the relatively small sample size constrains the ability to capture the full biological variability within the species. Third, the storage conditions applied in the study may not fully reflect commercial practices or consumer handling, potentially influencing the fatty acid profile of QE and QM.

## 5. Conclusions

The present study emphasizes the nutritional lipidomic diversity between quail eggs and meat, underlining their potential as functional foods. Greater awareness of the nutritional benefits of quail products is needed, particularly as they may serve as an important dietary alternative for supporting human health in developing countries. Furthermore, quail eggs and meat represent a promising substitute for chicken-derived foods, especially for consumers concerned with the ethical aspects of food production. Given the increasing demand for foods that are not only healthy and safe but also produced with respect for animal welfare, the comprehensive evaluation of quail products acquires particular relevance. In this context, quail-derived foods may justifiably be regarded as potential ‘superfoods’.

## Figures and Tables

**Table 1:** Profile of fatty acids (FAs) [ug/g sample] of eggs, breast, and thigh muscles of quails.

Sample		Eggs		Breast		Thigh		*p* Value of Two-Way ANOVA		
	Status	Fresh	Stored	Fresh	Stored	Fresh	Stored	Status	Sample	Interaction
Fatty Acids										
C6:0		20 ±14	21 ± 23	0.00 ± 0.00	0.00 ± 0.00	0.00 ± 0.00	0.00 ± 0.00	0.95	0.01	0.99
C8:0		20 ± 11	19 ± 10	8.2 ± 2.5	7.5 ± 2.4	10.21 ± 0.02	7.7 ± 2.1	0.65	0.02	0.97
C9:0		19.1 ± 2.6	24.6 ± 9.1	7.0 ± 1.8	7.2 ± 3.4	5.76 ± 0.10	10.7 ± 8.6	0.19	<0.001	0.67
C10:0		5.8 ± 1.2	13.2 ± 7.1	4.6 ± 2.1	3.83 ± 0.52	5.9 ± 1.7	5.49 ± 0.17	0.19	0.03	0.08
C11:0		18.1 ± 8.9	32 ± 14	6.9 ± 2.0	9.61 ± 0.02	8.3 ± 3.2	10.0 ± 7.0	0.11	<0.001	0.33
C12:0		15.4 ± 7.6	16.9 ± 6.9	12.5 ± 1.2	11.3 ± 8.6	10.62 ± 0.40	40 ± 52	0.36	0.57	0.44
C13:0		15.0 ± 3.3	22 ± 13	8.4 ± 3.8	7.4 ± 2.6	7.8 ± 3.9	7.3 ± 2.7	0.54	0.01	0.47
C14:0		227 ± 71	239 ± 60	34.0 ± 4.2	30.9 ± 7.3	23.5 ± 5.0	40 ± 29	0.66	<0.001	0.90
C15:0		22.4 ± 8.0	33.20 ± 0.20	8.92 ± 0.22	9.0 ± 1.5	8.4 ± 2.4	8.2 ± 2.0	0.05	<0.001	0.03
C16:0		5172 ± 943	5716 ± 1354	786 ± 37	633 ± 48	471 ± 31	624 ± 261	0.58	<0.001	0.68
C17:0		61 ± 24	77 ± 11	13.3 ± 5.5	18.2 ± 3.9	10.5 ± 1.6	17.9 ± 4.9	0.10	<0.001	0.67
C18:0		2142.00 ± 0.41	2357 ± 383	607 ± 44	541 ± 45	560 ± 11	639 ± 137	0.36	<0.001	0.38
C20:0		13.32 ± 0.92	13.9 ± 2.4	8.1 ± 3.6	8.7 ± 2.0	7.45 ± 0.72	7.6 ± 1.0	0.66	<0.001	0.98
C21:0		17.9 ± 1.4	26.9 ± 6.9	10.4 ± 1.7	8.07 ± 0.83	6.07 ± 1.1	7.6 ± 2.0	0.09	<0.001	0.02
C22:0		5.56 ± 0.63	10.53 ± 0.21	0.00 ± 0.00	0.00 ± 0.00	0.00 ± 0.00	0.00 ± 0.00	<0.001	<0.001	<0.001
C24:0		9.2 ± 3.2	13.0 ± 2.8	5.9 ± 1.2	4.8 ± 1.6	7.50 ± 0.98	6.80 ± 0.52	0.49	<0.001	0.09
ΣSFA		7644 ± 1250	8631 ± 1866	1520 ± 98	1303 ± 101	1145 ± 48	1443 ± 508	0.44	<0.001	0.56
c9C12:1		0.00 ± 0.00	0.00 ± 0.00	9.86 ± 0.03	8.3 ± 3.9	8.8 ± 5.01	9.5 ± 5.8	0.87	<0.001	0.86
c9 C14:1		60 ± 25	65 ± 26	6.1 ± 1.5	7.12 ± 1.3	7.9 ± 2.4	8.0 ± 3.6	0.76	<0.001	0.95
c7 C15:1		21.7 ± 3.6	24 ± 11	0.00 ± 0.00	0.00 ± 0.00	0.00 ± 0.00	0.00 ± 0.00	0.75	<0.001	0.90
c7 C16:1		275 ± 55	429 ± 216	25.3 ± 4.2	24.8 ± 6.2	15.2 ± 4.8	25 ± 15	0.23	<0.001	0.29
c9 C16:1		1628.0 ± 2.5	2166 ± 718	198 ± 31	148 ± 49	78 ± 14	139 ± 128	0.22	<0.001	0.23
c10 C16:1		14.1 ± 5.4	15.3 ± 4.2	0.00 ± 0.00	0.00 ± 0.00	0.00 ± 0.00	0.00 ± 0.00	0.75	<0.001	0.90
c10 C17:1		38.68 ± 0.25	49 ± 22	6.6 ± 3.1	7.8 ± 3.6	6.9 ± 3.5	7.3 ± 1.3	0.37	<0.001	0.60
t9 C18:1		61 ± 14	63.1 ± 6.9	8.3 ± 1.7	7.9 ± 1.7	7.4 ± 1.9	13.9 ± 1.5	0.42	<0.001	0.66
c9 C18:1 (OA)		7364 ± 1220	8561 ± 2584	905 ± 76	703 ± 187	354 ± 44	617 ± 439	0.47	<0.001	0.59
c11 C18:1		505.0 ± 3.4	713 ± 250	0.00 ± 0.00	0.00 ± 0.00	0.00 ± 0.00	0.00 ± 0.00	0.18	<0.001	0.17
c12 C18:1		50 ± 54	16.8 ± 7.2	0.00 ± 0.00	0.00 ± 0.00	0.00 ± 0.00	0.00 ± 0.00	0.31	0.03	0.36
c14 C18:1		8.8 ± 3.6	26 ± 11	0.00 ± 0.00	0.00 ± 0.00	0.00 ± 0.00	0.00 ± 0.00	0.02	<0.001	0.01
c11 C22:1		13.3 ± 6.2	14.9 ± 5.8	0.00 ± 0.00	0.00 ± 0.00	0.00 ± 0.00	0.00 ± 0.00	0.04	<0.001	0.02
c13 C22:1		5.8 ± 1.9	10.6 ± 3.1	0.00 ± 0.00	0.00 ± 0.00	0.00 ± 0.00	0.00 ± 0.00	0.75	<0.001	0.90
c15 C24:1		8.4 ± 3.6	14.7 ± 6.3	3.71 ± 0.59	5.51 ± 0.61	8.8 ± 4.5	7.4 ± 2.0	0.22	0.02	0.22
ΣMUFA		10635 ± 2216	12,168 ± 3832	1269 ± 126	988 ± 251	506 ± 0.67	897 ± 627	0.54	<0.001	0.69
c9c12 C16:2		12.5 ± 5.7	14.2 ± 3.4	11.1 ± 1.5	7.7 ± 5.0	9.44 ± 0.50	15.1 ± 5.3	0.51	0.26	0.20
c6c9c12 C16:3		0.00 ± 0.00	0.00 ± 0.00	12.4 ± 6.6	14.6 ± 2.5	12.91 ± 0.52	16.17 ± 0.31	0.21	<0.001	0.63
t9t12 C18:2		13.0 ± 5.7	10.3 ± 2.6	0.00 ± 0.00	0.00 ± 0.00	0.00 ± 0.00	0.00 ± 0.00	0.46	<0.001	0.58
c9c12 C18:2 (LA)		4855 ± 714	6123 ± 1624	1241 ± 198	1103 ± 278	711 ± 34	833 ± 149	0.25	<0.001	0.26
c6c9c12 C18:3 (GLA)		0.00 ± 0.00	0.00 ± 0.00	20 ± 12	7.2 ± 4.0	12.5 ± 4.3	17 ± 13	0.46	0.01	0.14
c8c11c14 C18:3		55.4 ± 11.3	96 ± 28	11.0 ± 4.0	5.5 ± 1.5	18.48 ± 0.08	13.0 ± 4.4	0.12	<0.001	0.01
c9c12c15 C18:3 (ALA)		942 ± 212	1608.4 ± 8.9	197 ± 29	136 ± 53	80.8 ± 7.6	94 ± 32	<0.001	<0.001	<0.001
c6c9c12c15 C18:4		14.58 ± 0.16	16.2 ± 8.9	0.00 ± 0.00	0.00 ± 0.00	0.00 ± 0.00	0.00 ± 0.00	0.75	<0.001	0.90
c9t11 C18:2 CLA		14.3 ± 1.7	16.6 ± 5.0	0.00 ± 0.00	0.00 ± 0.00	0.00 ± 0.00	0.00 ± 0.00	0.45	<0.001	0.56
c11t13 C18:2 CLA		19.7 ± 1.8	21.6 ± 3.7	11.0 ± 3.2	6.2 ± 2.4	8.1 ± 1.4	7.9 ± 2.5	0.42	<0.001	0.12
t10c12 C18:2 CLA		20.3 ± 5.3	25.3 ± 1.0	0.00 ± 0.00	0.00 ± 0.00	0.00 ± 0.00	0.00 ± 0.00	0.14	<0.001	0.12
c11c13 C18:2 CLA		31 ± 10	34.2 ± 5.2	0.00 ± 0.00	0.00 ± 0.00	0.00 ± 0.00	0.00 ± 0.00	0.67	<0.001	0.83
c11c14 C20:2		15.4 ± 3.5	13.9 ± 4.3	3.50 ± 0.56	3.91 ± 0.54	5.1 ± 1.0	3.3 ± 1.2	0.40	<0.001	0.69
c8c11c14 C20:3		66 ± 10	53 ± 14	13.29 ± 0.54	7.7 ± 1.8	12.5 ± 1.4	9.8 ± 5.3	0.06	<0.001	0.44
c5c8c11c14 C20:4 (AA)		632 ± 124	837 ± 217	417 ± 43	457 ± 32	249 ± 22	345 ± 29	0.04	<0.001	0.42
c11c14c17 C20:3		6.12 ± 0.63	14.1 ± 2.0	0.00 ± 0.00	0.00 ± 0.00	0.00 ± 0.00	0.00 ± 0.00	<0.001	<0.001	<0.001
c8c11c14c17 C20:4		0.00 ± 0.00	0.00 ± 0.00	6.4 ± 2.3	7.89 ± 0.49	6.55 ± 0.97	9.43 ± 0.74	0.01	<0.001	0.11
c5c8c11c14c17 C20:5 (EPA)		80 ± 15	85 ± 26	23.9 ± 2.2	22.1 ± 2.1	15.21 ± 1.34	16.5 ± 0.24	0.76	<0.001	0.87
c7c10c13c16c19 C22:5 (DPA)		98 ± 48	131 ± 26	37.4 ± 3.5	17.1 ± 3.2	40.96 ± 3.58	30.9 ± 5.7	0.94	<0.001	0.14
c4c7c10c13c16c19 C22:6 (DHA)		252 ± 208	658 ± 212	104.6 ± 4.7	211 ± 24	118 ± 14	241 ± 24	<0.001	<0.001	0.10
ΣPUFA		7142 ± 996	9563 ± 2419	2109 ± 294	2007 ± 287	1297 ± 72	1652 ± 255	0.11	<0.001	0.14
Total FA		25,421 ± 4151	30,362 ± 8081	4898 ± 514	4297 ± 559	2986 ± 139	3992 ± 1388	0.34	<0.001	0.45
Σn-3 PUFA		1397 ± 168	2304 ± 573	369 ± 37	393 ± 31	266 ± 19	392 ± 57	0.01	<0.001	0.02
Σn-6 PUFA		5690 ± 859	71,623 ± 1821	1717 ± 250	1594 ± 258	1008 ± 50	1231 ± 198	0.21	<0.001	0.26
∑n-3 PUFA/∑n-6 PUFA		0.25 ± 0.02	0.32 ± 0.01	0.22 ± 0.01	0.25 ± 0.02	0.26 ± 0.01	0.32 ± 0.01	<0.001	<0.001	0.06
ΣSFA/ΣPUFA		1.07 ± 0.12	0.91 ± 0.07	0.73 ± 0.06	0.66 ± 0.09	0.88 ± 0.06	0.86 ± 0.16	0.10	<0.001	0.53
ΣPUFA/ΣSFA		0.93 ± 0.06	1.11 ± 0.05	1.39 ± 0.04	1.54 ± 0.08	1.13 ± 0.04	1.14 ± 0.09	0.11	<0.001	0.55
ΣSFA/ΣUFA		0.43 ± 0.01	0.40 ± 0.04	0.45 ± 0.03	0.44 ± 0.08	0.62 ± 0.02	0.57 ± 0.01	0.12	<0.001	0.57

ΣSFA—sum of saturated fatty acids; OA—oleic acid; ΣMUFA—sum of monounsaturated fatty acids; LA—linoleic acid; ALA—α-linolenic acid; CLA—conjugated linoleic acid; AA—arachidonic acid; EPA—eicosapentaenoic acid; DPA—docosapentaenoic acid; DHA—docosahexaenoic acid; ΣPUFA—sum of polyunsaturated fatty acids; Total FA—total sum of all detected fatty acids; Σn-3 PUFA—sum of polyunsaturated fatty acids of the n-3 family; Σn-6 PUFA—sum of polyunsaturated fatty acids of the n-6 family; Σn-3 PUFA/Σn-6 PUFA—ratio of n-3 polyunsaturated fatty acids to the n-6 fatty acids; ΣSFA/ΣPUFA—ratio of sum of saturated fatty acids to the sum of the polyunsaturated fatty acids; ΣPUFA/ΣSFA—ratio of sum of polyunsaturated fatty acids to the sum of the saturated fatty acids; ΣSFA/ΣUFA—ratio of the sum of saturated fatty acids to the sum of unsaturated fatty acids.

**Table 2:** Loadings, eigenvalues, and variances of the principal components (PCs).

Variables	PC1	PC2
C12:0	−0.028	**0.695**
C14:0	**−0.969**	0.002
C15:0	**−0.940**	−0.058
C16:0	**−0.989**	−0.074
C17:0	**−0.963**	−0.056
C18:0	**−0.984**	−0.048
ΣSFA	**−0.993**	−0.047
c9 C14:1	**−0.953**	0.017
c7 C16:1	**−0.974**	0.067
c9 C16:1	**−0.990**	−0.026
c10 C17:1	**−0.972**	0.026
t9 C18:1	**−0.967**	−0.064
c9 C18:1 (OA)	**−0.992**	−0.067
ΣMUFA	**−0.989**	−0.057
c9c12 C16:2	−0.425	**0.602**
c9c12 C18:2 (LA)	**−0.992**	−0.085
c8c11c14 C18:3	**−0.958**	0.107
c9c12c15 C18:3 (ALA)	**−0.962**	−0.071
c11t13 C18:2 CLA	**−0.943**	−0.098
c5c8c11c14c17 C20:5 (EPA)	**−0.919**	−0.284
c7c10c13c16c19 C22:5 (DPA)	**−0.923**	−0.035
ΣPUFA	**−0.993**	−0.061
Total FA	**−0.995**	−0.055
Σn-3 PUFA	**−0.982**	0.022
Σn-6 PUFA	**−0.991**	−0.089
Σn-3 PUFA/Σn-6 PUFA	−0.358	**0.602**
Eigenvalue	33.6	
Variance (%)	75.4	6.5
Cumulative (%)	81.9	

(The most significant loadings are boldfaced). ΣSFA—sum of saturated fatty acids; OA—oleic acid; ΣMUFA—sum of monounsaturated fatty acids; LA—linoleic acid; ALA—α-linolenic acid; CLA—conjugated linoleic acid; EPA—eicosapentaenoic acid; DPA—docosapentaenoic acid; ΣPUFA—sum of polyunsaturated fatty acids; Total FA—total sum of all detected fatty acids; Σn-3 PUFA—sum of polyunsaturated fatty acids of the n-3 family; Σn-6 PUFA—sum of polyunsaturated fatty acids of the n-6 family; Σn-3 PUFA/Σn-6 PUFA—ratio of n-3 polyunsaturated fatty acids to the n-6 polyunsaturated fatty acids.

## Data Availability

Data is contained within the article and Supplementary Materials.

## Supplementary Materials

The following supporting information can be downloaded at https://www.mdpi.com/article/10.3390/molecules31030407/s1, Table S1: Nutritional composition and fatty acids profile [per g of sample] of quails’ feed.

## Abbreviations

The following abbreviations are used in this manuscript:

AA	arachidonic acid
ALA	alpha linolenic acid
ANOVA	analysis of variance
B	breast
c	cis
CA	cluster analysis
CL	cluster
DHA	docosahexaenoic acid
EPA	eicosapentaenoic acid
EU	European Union
FA	fatty acid
FAME	fatty acids methyl esters
FAOSTAT	Food and Agriculture Organization Corporate Statistical Database
GC-MS	gas chromatography—mass spectrometry
GLA	gamma linolenic acid
JQ	Japanese quail
LA	linoleic acid
MUFA	monounsaturated fatty acids
OA	oleic acid
PC	principal component
PCA	Principal Component Analysis
PUFA	polyunsaturated fatty acids
QE	quail eggs
QM	quail meat
SD	standard deviation
SFA	saturated fatty acids
t	trans
T	thigh
UFA	unsaturated fatty acids
