# Supplementary material for: Lipidomic Insight into Eggs and Meat of Quail (Coturnix japonica) as Potential ‘Superfoods’
Source: Molecules. 2026 Jan 24;31(3):407. doi: 10.3390/molecules31030407 (PMC12898336; doi:10.3390/molecules31030407)
Supplement: Supplementary file 1 [file molecules-31-00407-s001.zip › molecules-4100460-supplementary.pdf]

Table S1. Nutritional composition and fatty acids profile [per g of sample] of quails′ feed[a].

**Nutrients [per kg of diet]**	
Crude protein [%]	21
Crude fat [%]	4.93
Crude fiber [%]	3.5
Crude ash [%]	9.67
Lysine [%]	1.15
Methionine [%]	0.45
Calcium [%]	2.34
Phosphorus [%]	0.14
Available phosphorus [%]	0.52
Sodium [%]	0.14
Vitamin A [IU/kg]	15000
Vitamin D3 [IU/kg]	3125
Iodine [mg]	1
Zinc [mg]	70
Manganese [mg]	85
Selenium [mg]	0.20
**Fatty acids [mg/g of sample]**	
C8:0	5.0±1.7
C9:0	11.9±2.8
C10:0	3.25±0.28
C11:0	11.9±2.2
C12:0	21.7±5.9
C13:0	3.7±1.5
C14:0	27.7±3.4
C15:0	7.6±3.0
C16:0	1796±461
C17:0	15.4±4.9
C18:0	568±134
C20:0	13.9±2.7
C21:0	3.58±0.68
C24:0	6.5±1.7
Σ SFA	2496±612
c9 C14:1	6.2±1.3
c7 C16:1	14.8±5.0
c9 C16:1	40±12
c10 C17:1	7.6±1.5
c9 C18:1 (OA)	2866±660
c11 C18:1	132±32
c14 C18:1	10.1±4.5
Σ MUFA	3076±715
c9c12 C18:2 (LA)	9038±2291
c6c9c12 C18:3 (GLA)	5.1±3.3
c9c12c15 C18:3 (ALA)	5034±891
c11c13 C18:2 CLA	16.6±3.4
c6c9c12c15 C18:4	5.2±3.0
c5c8c11c14 C20:4 (AA)	3.2±1.3
c8c11c14c17 C20:4	11.7±3.6
c6c9c12c15c18c21 C24:6	8.6±9.4

Σ PUFA	14123±3144
Total FA	19695±4447
Σ n-3 PUFA	5048±882
Σ n-6 PUFA	9075±2302

[a] ingredients: corn, soybean meal, wheat, triticale, rapeseed meal, rapeseed, calcium carbonate, wheat gluten feed, dried red blood cells, dicalcium phosphate, vegetable oil, sodium chloride; SFA – sum of saturated fatty acids; OA – oleic acid; MUFA – sum of monounsaturated fatty acids; LA – linoleic acid; ALA – α-linolenic acid; CLA – conjugated linoleic acid; AA – arachidonic acid; Σ PUFA – sum of polyunsaturated fatty acids; total FA – total sum of all detected fatty acids; Σ n-3 PUFA – sum of polyunsaturated fatty acids of the n-3 family; Σ n-6 PUFA – sum of polyunsaturated fatty acids of the n-6 family.
